# Supplementary material for: COVID-19 Vaccine Acceptability and Its Determinants in Mozambique: An Online Survey
Source: Vaccines (Basel). 2021 Jul 27;9(8):828. doi: 10.3390/vaccines9080828 (PMC8402577; doi:10.3390/vaccines9080828)
Supplement: Supplementary file 1 [file vaccines-09-00828-s001.zip › File S1.pdf]

Moçambique vacinação COVID-19

IDENTIFICAÇÃO DO PARTICIPANTE

A COVID-19 é uma doença respiratória aguda que se manifesta por um quadro de gripe na fase ligeira mas pode tornar-se grave com dificuldade respiratória e risco de morte. Este é um problema de saúde mundial que se propagou para Moçambique. Neste momento em Moçambique, registrou-se 62.520 casos e 693 óbitos de COVID-19.

A transmissão de COVID-19 ocorre entre humanos por via respiratória, objectos contaminados e contacto físico directo com pessoas infectadas.

As medidas implementadas para prevenção de COVID-19 incluem a combinação: distanciamento físico entre as pessoas, uso de mascaras faciais, lavagem e/ou desinfecção frequente das mãos e objectos, e actualmente a vacinação contra a COVID-19. Com este questionário pretendemos entender a aceitabilidade da vacina contra COVID-19 em Moçambique.

Convidamos os voluntários a preencher as suas opiniões neste questionário o mais fielmente possível; Em geral responder ao questionário leva em media 10 a 15 minutos.

A sua participação deve ser voluntária. O participante deve ter idade igual ou superior a 18 anos. Você pode desistir de participar a qualquer momento, sem qualquer represálias. Seus dados serão anónimos e tratados confidencialmente. Não prevemos ocorrência de riscos ao participar desta pesquisa, e nem benefícios individuais; os resultados ajudarão a evidenciar a aceitação e preocupações da população com relação a vacinação contra COVID-19. Precisando de mais informações poderá contactar os pesquisadores e consultar as nossas medidas de confidencialidade no site www.ICPCovid.com.

Caso tenha qualquer dúvida relacionada a este inquérito, por favor pode contactar a equipa de pesquisa, através do email: **psistemasdesaude@gmail.com**. Contactos: 822718644 (Comité Institucional de Bioética para a Saúde) ou 877653701 (Investigadora Principal).

Q: Expressão de Consentimento: (*)

Type: choice

A: one of the following:

Sim	=>	*Aceito participar*
Não	=>	*Não Aceito participar*

INFORMAÇÃO DO INTREVISTADO

Q: Província (*)

Type: choice

A: one of the following:

Gaza	=>	*Gaza*
Inhambane	=>	*Inhambane*
Cabo_Delgado	=>	*Cabo Delgado*
Manica	=>	*Manica*
Maputo_cidade	=>	*Maputo (cidade)*
Maputo	=>	*Maputo*
Nampula	=>	*Nampula*
Niassa	=>	*Niassa*
Sofala	=>	*Sofala*
Tete	=>	*Tete*

Zambézia	=>	*Zambézia*

Q: Gaza (*)

Type: choice

A: one of the following:

Bilene	=>	*Bilene*
Chibuto	=>	*Chibuto*
Chicualacuala	=>	*Chicualacuala*
Chigubo	=>	*Chigubo*
Chockwe	=>	*Chockwe*
Chongoene	=>	*Chongoene*
Guijá	=>	*Guijá*
Limpopo	=>	*Limpopo*
Mabalane	=>	*Mabalane*
Mandlakazi	=>	*Mandlakazi*
Mapai	=>	*Mapai*
Massangena	=>	*Massangena*
Massingir	=>	*Massingir*
Xai-Xai	=>	*Xai-Xai*

Q: Inhambane (*)

Type: choice

A: one of the following:

Inhambane	=>	*Inhambane*
Funhalouro	=>	*Funhalouro*
Govuro	=>	*Govuro*
Homoine	=>	*Homoine*
Inharrime	=>	*Inharrime*
Inhassoro	=>	*Inhassoro*
Jangamo	=>	*Jangamo*
Mabote	=>	*Mabote*
Massinga	=>	*Massinga*
Maxixe	=>	*Maxixe*
Morrumbene	=>	*Morrumbene*
Panda	=>	*Panda*
Vilanculos	=>	*Vilanculos*
Zavala	=>	*Zavala*

Q: Cabo Delgado (*)

Type: choice

A: one of the following:

Ancuabe	=>	*Ancuabe*
Balama	=>	*Balama*

Chiure	=>	*Chiure*
Ibo	=>	*Ibo*
Macomia	=>	*Macomia*
Mecufi	=>	*Mecufi*
Meluco	=>	*Meluco*
Metuge	=>	*Metuge*
Mocimboa_da_Praia	=>	*Mocimboa da Praia*
Montepuez	=>	*Montepuez*
Mueda	=>	*Mueda*
Muidumbe	=>	*Muidumbe*
Namuno	=>	*Namuno*
Nangade	=>	*Nangade*
Palma	=>	*Palma*
Pemba	=>	*Pemba*
Quissanga	=>	*Quissanga*

Q: Manica (*)

Type: choice

A: one of the following:

Manica	=>	*Manica*
Bárue	=>	*Bárue*
Chimoio	=>	*Chimoio*
Gondola	=>	*Gondola*
Guro	=>	*Guro*
Macate	=>	*Macate*
Machaze	=>	*Machaze*
Macossa	=>	*Macossa*
Mossurize	=>	*Mossurize*
Sussundenga	=>	*Sussundenga*
Tambara	=>	*Tambara*
Vanduzi	=>	*Vanduzi*

Q: Maputo Cidade (*)

Type: choice

A: one of the following:

Kamavota	=>	*Kamavota*
Kamaxakeni	=>	*Kamaxakeni*
Kamubukwane	=>	*Kamubukwane*
Kanyaka	=>	*Kanyaka*
Katembe	=>	*Katembe*
Khampfumo	=>	*Khampfumo*
Nlhamankulo	=>	*Nlhamankulo*

Q: Maputo (*)

Type: choice

A: one of the following:

Boane	=>	*Boane*
Magude	=>	*Magude*
Manhiça	=>	*Manhiça*
Marracuene	=>	*Marracuene*
Matola	=>	*Matola*
Matutine	=>	*Matutine*
Moamba	=>	*Moamba*
Namaacha	=>	*Namaacha*

Q: Nampula (*)

Type: choice

A: one of the following:

Angoche	=>	*Angoche*
Nampula	=>	*Nampula*
Erati	=>	*Erati*
Ilha_de_Moçambique	=>	*Ilha de Moçambique*
Lalaua	=>	*Lalaua*
Larde	=>	*Larde*
Liupo	=>	*Liupo*
Malema	=>	*Malema*
Meconta	=>	*Meconta*
Mecuburi	=>	*Mecuburi*
Memba	=>	*Memba*
Mogincual	=>	*Mogincual*
Mogovolas	=>	*Mogovolas*
Moma	=>	*Moma*
Monapo	=>	*Monapo*
Mossuril	=>	*Mossuril*
Muecate	=>	*Muecate*
Murrupula	=>	*Murrupula*
Nacala_Porto	=>	*Nacala Porto*
Nacala-a-velha	=>	*Nacala-a-velha*
Nacaroa	=>	*Nacaroa*
Rapale	=>	*Rapale*
Ribaue	=>	*Ribaue*

Q: Niassa (*)

Type: choice

A: one of the following:

Chimbonila	=>	*Chimbonila*

Cuamba	=>	*Cuamba*
Lago	=>	*Lago*
Majune	=>	*Ilha de Moçambique*
Mandimba	=>	*Mandimba*
Marrupa	=>	*Marrupa*
Maua	=>	*Maua*
Mavago	=>	*Mavago*
Mecanhelas	=>	*Mecanhelas*
Mecula	=>	*Mecula*
Metarica	=>	*Metarica*
Mueda	=>	*Mueda*
Muembe	=>	*Muembe*
Ngauma	=>	*Ngauma*
Nipepe	=>	*Nipepe*
Sanga	=>	*Sanga*

Q: Sofala (*)

Type: choice

A: one of the following:

Beira	=>	*Beira*
Buzi	=>	*Buzi*
Caia	=>	*Caia*
Chemba	=>	*Chemba*
Cheringoma	=>	*Cheringoma*
Chibabava	=>	*Chibabava*
Dondo	=>	*Dondo*
Gorongoza	=>	*Gorongoza*
Machanga	=>	*Machanga*
Maringue	=>	*Maringue*
Marromeu	=>	*Marromeu*
Muanza	=>	*Muanza*
Nhamatanda	=>	*Nhamatanda*

Q: Tete (*)

Type: choice

A: one of the following:

Tete	=>	*Tete*
Angónia	=>	*Angónia*
Cahora_Bassa	=>	*Cahora Bassa*
Changara	=>	*Changara*
Chifunde	=>	*Chifunde*
Chiuta	=>	*Chiuta*
Doa	=>	*Doa*

Macanga	=>	*Macanga*
Magoe	=>	*Magoe*
Marara	=>	*Marara*
Marávia	=>	*Marávia*
Moatize	=>	*Moatize*
Mutarara	=>	*Mutarara*
Tsangano	=>	*Tsangano*
Zumbo	=>	*Zumbo*

Q: Zambezia (*)

Type: choice

A: one of the following:

Alto_Molocue	=>	*Alto Molocue*
Chinde	=>	*Chinde*
Derre	=>	*Derre*
Gilé	=>	*Gilé*
Gurué	=>	*Gurué*
Ile	=>	*Ile*
Inhassunge	=>	*Inhassunge*
Luabo	=>	*Luabo*
Lugela	=>	*Lugela*
Maganja_da_Costa	=>	*Maganja da Costa*
Milange	=>	*Milange*
Mocuba	=>	*Mocuba*
Mocubela	=>	*Mocubela*
Molumbo	=>	*Molumbo*
Mopeia	=>	*Mopeia*
Morrumbala	=>	*Morrumbala*
Mulevala	=>	*Mulevala*
Namacurra	=>	*Namacurra*
Namarroi	=>	*Namarroi*
Nicoadala	=>	*Nicoadala*
Pebane	=>	*Pebane*
Quelimane	=>	*Quelimane*

Q: É profissional de saúde? (*)

Type: choice

A: one of the following:

Sim	=>	*Sim*
Não	=>	*Não*

Q: Em que tipo de instituição trabalha? (*)

Type: choice

A: one of the following:

Misau	=>	*Misau*
DPS/SPAS/DDS	=>	*DPS/SPAS/DDS*
Instituição de pesquisa	=>	*Instituição de pesquisa*
Instituição de formação	=>	*Instituição de formação*
ONG/OSC	=>	*ONG/OSC*
Hospital Provincial	=>	*Hospital Provincial*
Outro	=>	*Outro*
Unidade Sanitária	=>	*Unidade Sanitária*

Q: Tipo de Unidade Sanitária

Type: choice

A: one of the following:

CS_Rural	=>	*Centro de Saúde Rural*
CS_Urbano	=>	*Centro de Saúde Urbano*
HD	=>	*Hospital Distrital*
HG	=>	*Hospital Geral*
HR	=>	*Hospital Rural*
HP	=>	*Hospital Provincial*
HC	=>	*Hospital Central*

Visible if

Q:	A:
É profissional de saúde?	- value => Sim
Em que tipo de instituição trabalha?	- value => Unidade Sanitária

Q: Formação

Type: choice

A: one of the following:

Enfermagem	=>	*Enfermagem*
Médico(a)	=>	*Médico(a)*
Agente_de_Serviço	=>	*Agente de Serviço*
Técnico_de_Saúde	=>	*Técnico de Saúde*
Funcionário_da_área_administrativa	=>	*Funcionário da área administrativa*
Outra	=>	*Outra, especificar*

Q: Especifique (*)

Type: text

A: text input

Visible if

Q:	A:
Formação	- value => Outra

Q: Função que ocupa

Type: choice

A: one of the following:

Atendimento_clinico	=>	*Atendimento clinico*
Laboratorial	=>	*Laboratorial*
Saúde_Publica	=>	*Saúde Publica*
Área_administrativa	=>	*Área administrativa*
Agentes_de_Serviços	=>	*Agentes de Serviços*
Pesquisador	=>	*Pesquisador*
Outra	=>	*Outra*

Q: Especificar (*)

Type: text

A: text input

Visible if

Q:	A:
Função que ocupa	- value => Outra

Q: A que grupo pertence (*)

Type: choice

A: one of the following:

Doente_crónico	=>	*Doente crónico*
Gestante	=>	*Gestante*
Forças_Armadas	=>	*Forças de defesa e segurança*
Idoso	=>	*Idoso vivendo em lar para idosos*
Professores	=>	*Professores*
Nenhum	=>	*Nenhum*

Q: Bairro de residência (*)

Type: text

A: text input

Q: Zona de residência: (*)

Type: choice

A: one of the following:

Zona_Urbana	=>	*Zona Urbana*
Zona_Rural	=>	*Zona Rural*
Zona_Periurbana	=>	*Zona Periurbana*
Vila	=>	*Vila*
Aldeia	=>	*Aldeia*

Q: Idade (*)

Type: number

A: number (min: 18 / max: 110 / step: 1)

Q: Sexo (*)

Type: choice

A: one of the following:

male	=>	*Masculino*
female	=>	*Feminino*

Q: Estado Civil (*)

Type: choice

A: one of the following:

Casado(a)	=>	*Casado(a)*
Solteiro(o)	=>	*Solteiro(o)*
Vive_maritalmente	=>	*Vive maritalmente*
Divorciado(a)	=>	*Divorciado(a)*
Viúvo(a)	=>	*Viúvo(a)*

Q: Afiliação Religiosa (*)

Type: choice

A: one of the following:

Muçulmana	=>	*Muçulmana*
Zione	=>	*Zione*
Cristã	=>	*Cristã*
Sem_religião	=>	*Sem religião*
Outra	=>	*Outra*

Q: Qual é a sua ocupação? (*)

Type: choice

A: one of the following:

Estudante	=>	*Estudante*
Trabalhador	=>	*Trabalhador*
Outro	=>	*Outro, especificar*

Q: Especificar (*)

Type: text

A: text input

Visible if

Q:	A:
Qual é a sua ocupação?	- value => Outro

Q: Se for estudante, por favor selecciona o seu nível de escolaridade (*)

Type: choice

A: one of the following:

Primário	=>	*Primário*
Secundário	=>	*Secundário*
Graduado universitária	=>	*Graduado universitária*
Pós-graduação universitária	=>	*Pós-graduação universitária*

Q: Se for trabalhador, por favor selecciona o seu sector (*)

Type: choice

A: one of the following:

Sector_de_Educação	=>	*Sector de Educação*
Sector_de_Saúde	=>	*Sector de Saúde*
Sector_de_Agricultura	=>	*Sector de Agricultura*
Sector_das_Pescas	=>	*Sector das Pescas*
Sector_de_Economia_e_Finanças	=>	*Sector de Economia e Finanças*
Sector_de_Indústria_e_Comércio	=>	*Sector de Indústria e Comércio*
Sector_Privado	=>	*Sector Privado*
Outro	=>	*Outro*

Q: Com quem você mora actualmente na mesma casa? (*)

Type: choice_multiple

A: multiple answers possible:

Pai	=>	*Pai*
Mãe	=>	*Mãe*
Esposo_(a)	=>	*Esposo (a)*
Filhos	=>	*Filhos*

Tia(a)	=>	*Tia(a)*
Irmão(a)	=>	*Irmão(a)*
Cunhado(a)	=>	*Cunhado(a)*
Sogro(a)	=>	*Sogro(a)*
Sobrinho(a)	=>	*Sobrinho(a)*
Trabalhador_domestico	=>	*Trabalhador doméstico*
Sozinho	=>	*Sozinho*

Q: Selecione o número de pessoas em cada categoria de idade. (*)

Type: choice_scale

A: 60+18 a 59 anos12 a 17 anosMenores de 12 Anos

0	=>	*0*
1	=>	*1*
2	=>	*2*
3	=>	*3*
4	=>	*4*
5	=>	*5*
6	=>	*6*
7	=>	*7*
8	=>	*8*
9	=>	*9*
10	=>	*10*

Q: Qual das seguintes categorias descreve melhor sua situação socioeconómica actual considerando o valor mensal de referencia de 30 mil meticais? (*)

Type: choice

A: one of the following:

muito_baixa_em_relação_a_30 mil_meticais	=>	*muito baixa em relação a 30 mil meticais*
baixa_em_relação_a_30_mil_meticais	=>	*baixa em relação a 30 mil meticais*
em_torno_de_30_mil_meticais	=>	*em torno de 30 mil meticais*
acima_de _30_mil_meticais	=>	*acima de 30 mil meticais*
Muito_acima_de_30_mil_meticais	=>	*Muito acima de 30 mil meticais*
Não_tenho_rendimento	=>	*Não tenho rendimento*

SAÚDE E HISTÓRIA DO COVID-19

Q: Já ouviu falar do coronavírus/covid-19? (*)

Type: choice

A: one of the following:

Sim	=>	*Sim*
Não	=>	*Não*

Q: Quais são os sintomas mais comuns da covid-19 (o que ela faz/provoca)? (*)

Type: choice_multiple

A: multiple answers possible:

Febres	=>	*Febres*
Dor_de_Cabeça	=>	*Dor de Cabeça*
Dores_nas_articulações	=>	*Dores nas articulações*
Dificuldades_em_respirar	=>	*Dificuldades em respirar*
Tosse_seca	=>	*Tosse seca*
Cansaço	=>	*Cansaço*
Outros	=>	*Outros*

Visible if

Q:	A:
Já ouviu falar do coronavírus/covid-19?	- value => Sim

Q: Como pode se contrair a covid-19? (*)

Type: choice_multiple

A: multiple answers possible:

Abraço	=>	*Abraço*
Beijo	=>	*Beijo*
Aperto_de_mão	=>	*Aperto de mão*
Ficar_em_aglomerados	=>	*Ficar em aglomerados*
Outros	=>	*Outros*

Visible if

Q:	A:
Já ouviu falar do coronavírus/covid-19?	- value => Sim

Q: Desde o início da pandemia você testou para covid-19? (*)

Type: choice

A: one of the following:

Sim	=>	*Sim*
Não	=>	*Não*

Visible if

Q:	A:
Já ouviu falar do coronavírus/covid-19?	- value => Sim

Q: Se sim, qual foi o resultado do seu estado de saúde (*)

Type: choice

A: one of the following:

Eu_fui_testado_positivo	=>	*Eu fui testado positivo*
Eu_fui_testado_negativo	=>	*Eu fui testado negativo*
Não_sei_os_resultados_do_meu_teste	=>	*Não sei os resultados do meu teste*

Visible if

Q:	A:
Desde o início da pandemia você testou para covid-19?	- value => Sim
Já ouviu falar do coronavírus/covid-19?	- value => Sim

Q: Quanto você está preocupado / com medo de se tornar (re) infectado pelo coronavírus?(*)

Type: choice_scale

A: Em uma escala de 1 a 5

1	=>	*1=Nada preocupado*
2	=>	*2=Um pouco preocupado*
3	=>	*3=Moderadamente preocupado*
4	=>	*4=Muito preocupado*
5	=>	*5=Extremamente preocupado*

Visible if

Q:	A:
Já ouviu falar do coronavírus/covid-19?	- value => Sim

Q: Você sofre das seguintes condições crónicas de saúde? (muitas respostas possíveis) (*)

Type: choice_multiple

A: multiple answers possible:

Doença_cardíaca	=>	*Doença cardíaca*
Hipertensão_(Tensão)	=>	*Hipertensão (Tensão)*
Câncer	=>	*Câncer*
HIV	=>	*HIV*
Tuberculose	=>	*Tuberculose*
Doenças_respiratórias	=>	*Doenças respiratórias*
AVC	=>	*AVC*
Não_sofro_de_doença_crónica	=>	*Não sofro de doença crónica*
Outro	=>	*Outro*

Q: Nos últimos 7 dias, você implementou qual das medidas preventivas contra a COVID-19? (marque todas as respostas que se aplicam) (*)

Type: choice_multiple

A: multiple answers possible:

Distanciamento_físico_de_pelo_menos_1,5m	=>	*Distanciamento físico de pelo menos 1,5m*
Uso_máscara_facial	=>	*Uso máscara facial*
Higiene_das_mãos	=>	*Higiene das mãos (lavagem regular das mãos com sabão ou álcool-gel para as mãos ou cinza)*
Etiqueta_da_tosse	=>	*Etiqueta da tosse (cobrir a boca ao tossir ou espirrar)*
• Nenhuma_das_opções_acima	=>	*Nenhuma das opções acima*

Nos últimos 15 dias, quanto sentiu os seguintes sintomas?

Q: Sintomas (*)

Type: choice_scale

A: Sentiu-se para baixo, deprimido ou sem esperança?Pouco interesse ou prazer em realizar tarefas/actividades?Não ser capaz de parar ou controlar as preocupaçõesSentiu-se nervoso, ansioso ou no limite/esgotado

1	=>	*1=De modo nenhum*
2	=>	*2=Poucos dias*
3	=>	*3=Menos da metade dos dias*
4	=>	*4=Mais da metade dos dias*
5	=>	*5=Quase todos os dias*

COVID-19 VACINAÇÃO

Q: Na sua opinião, alguém pode ser infectado com coronavírus após a recuperação de uma infecção anterior por COVID-19? (*)

Type: choice

A: one of the following:

Sim	=>	*Sim*
Não	=>	*Não*
Eu não sei	=>	*Eu não sei*

Q: Na sua opinião, a infecção por COVID-19 pode ser prevenida pela vacina actualmente disponível em Moçambique?(*)

Type: choice

A: one of the following:

Sim	=>	*Sim*
Não	=>	*Não*
Eu não sei	=>	*Eu não sei*

Q: No seu melhor conhecimento, há actualmente uma vacina eficaz contra covid-19 Mundo? (*)

Type: choice

A: one of the following:

Sim	=>	*Sim*
Não	=>	*Não*
Eu não sei	=>	*Eu não sei*

Q: Você estaria disposto a ser administrado(a) alguma das vacinas existentes contra COVID-19? (*)

Type: choice

A: one of the following:

Sim	=>	*Sim*
Não	=>	*Não*

Q: O que levaria em conta para ser administrado(a) a vacina contra Covid-19 ?(*)

Type: choice

A: one of the following:

Origem_da_vacina	=>	*Origem da vacina*
Marca_da_Vacina	=>	*Marca da Vacina*
Outros	=>	*Outros Aspectos*
Nenhuma	=>	*Nenhuma*

Visible if

Q:	A:
Você estaria disposto a ser administrado(a) alguma das vacinas existentes contra COVID-19?	- value => Sim

Q: Origem da vacina, aliste (*)

Type: choice_multiple

A: multiple answers possible:

Alemanhã	=>	*Alemanhã*
EUA	=>	*Estados Unidos da América*
Rússia	=>	*Rússia*
Reino_Unido	=>	*Reino Unido*
China	=>	*China*
Índia	=>	*Índia*
Não sei	=>	*Não sei*

Visible if

Q:	A:
O que levaria em conta para ser administrado(a) a vacina contra Covid-19 ?	- value => Origem_da_vacina

Q: Marca da vacina, aliste (*)

Type: choice_multiple

A: multiple answers possible:

Pfizer-BioNTech	=>	*Pfizer-BioNTech fosun farma*
Moderna	=>	*Moderna*
Sputnik-V/Gameleya	=>	*Sputnik-V/Gameleya*
ChAdOx1-S_nCov19_Astrazeneca	=>	*ChAdOx1-S nCov19 da Astrazeneca-Universidade de Oxford*
CoronaVac_(Sinovac)	=>	*CoronaVac_(Sinovac)*
CanSino	=>	*CanSino*
Johnson_&_Johnson	=>	*Johnson & Johnson*
Sinopharm	=>	*Sinopharm*
Bharat_Biotech	=>	*Bharat Biotech*
Novavax_(NVX-CoV2373)	=>	*Novavax (NVX-CoV2373)*
Anhui_Zhifei_Longcom	=>	*Anhui Zhifei Longcom - RBD-Dmer*
Não sei	=>	*Não sei*

Visible if

Q:	A:
O que levaria em conta para ser administrado(a) a vacina contra Covid-19 ?	- value => Marca_da_Vacina

Q: Eficacia na prevenção da infecção (*)

Type: choice_scale

A: Se for pelo menos 50% a 60% eficaz na prevenção da infecçãoSe for pelo menos 60% a 70% eficaz na prevenção da infecçãoSe for pelo menos 70% a 80% a eficaz na prevenção da infecçãoSe for pelo menos 80% a 90% eficaz na prevenção da infecçãoSe for pelo menos 90%+ eficaz na prevenção da infecção

1	=>	*1=Sim*
2	=>	*2=Não*
3	=>	*3=Não Tenho Opinião*

Visible if

Q:	A:
Você estaria disposto a ser administrado(a) alguma das vacinas existentes contra COVID-19?	- value => Sim

Q: Qual seria a técnica de vacinação da sua preferência (*)

Type: choice

A: one of the following:

Seringas	=>	*Seringas*
Vacina_Oral	=>	*Vacina Oral*

Visible if

Q:	A:
Você estaria disposto a ser administrado(a) alguma das vacinas existentes contra COVID-19?	- value => Sim

Q: Seringas Porquê? (*)

Type: choice_multiple

A: multiple answers possible:

Segura	=>	*Segura*
Eficiente	=>	*Eficiente*
Outro	=>	*Outro*

Visible if

Q:	A:
Qual seria a técnica de vacinação da sua preferência	- value => Seringas

Q: Vacina Oral Porquê? (*)

Type: choice_multiple

A: multiple answers possible:

Segura	=>	*Segura*
Eficiente	=>	*Eficiente*
Outro	=>	*Outro*

Visible if

Q:	A:
Qual seria a técnica de vacinação da sua preferência	- value => Vacina_Oral

Q: Quais são os possíveis motivos pelo qual você hesitaria em tomar a vacina de COVID-19? (muitas respostas possíveis) (*)

Type: choice_multiple

A: multiple answers possible:

COVID-19_Nao_Existe	=>	*Eu não acho que COVID-19 existe*
Nao_Eficaz	=>	*Eu acho que a vacina não é eficaz*
Prejudica	=>	*Eu acho que a vacina foi feita para nos prejudicar*
Medo_de_Efeitos_Colaterais	=>	*Estou com medo dos efeitos colaterais da vacina*
Corpo_Naturalmente_Forte	=>	*Meu corpo é naturalmente forte, não preciso de uma vacina para lutar contra COVID-19*
Doenças_respiratórias	=>	*Doenças respiratórias*
Tinha_COVID_Sou_Imune	=>	*Eu já tinha COVID-19, então acho que sou imune à doença*
A_COVID_terminou_na_minha_Provincia	=>	*A pandemia COVID-19 terminou na minha província, não há necessidade de vacina agora*
Nenhuma_das_opções_acima	=>	*Nenhuma das opções acima*
Outros	=>	*Outros Motivos*

Q: Importancia da Vacinação (*)

Type: choice_scale

A: Vacinar-se contra COVID-19, é importante para proteger a sua saúde?Vacinar-se contra COVID-19, é algo importante para proteger outras pessoas na sua comunidade?

1	=>	*1=Nada importante*
2	=>	*2=Um pouco importante*
3	=>	*3=Moderadamente importante*
4	=>	*4=Muito importante*
5	=>	*5=Extremamente importante*

Q: Experiências passadas com vacinação? (*)

Type: choice

A: one of the following:

Positiva	=>	*Positiva*
Negativa	=>	*Negativa*

Q: Positiva Porquê? (*)

Type: choice_multiple

A: multiple answers possible:

Qualidade_do_Provedor	=>	*Qualidade do Provedor*
Organização_do_Servico_de_Saude	=>	*Organização do Servico de Saude*
Segurança_da_vacina	=>	*Segurança da vacina*
Eficácia_da_vacina	=>	*Eficácia da vacina*
Outros	=>	*Outros, especifique*
Sem_Opinião	=>	*Sem Opinião*

Visible if

Q:	A:
Experiências passadas com vacinação?	- value => Positiva

Q: Negativa Porquê? (*)

Type: choice_multiple

A: multiple answers possible:

Qualidade_do_Provedor	=>	*Qualidade do Provedor*
Organização_do_Servico_de_Saude	=>	*Organização do Servico de Saude*
Segurança_da_vacina	=>	*Segurança da vacina*
Eficácia_da_vacina	=>	*Eficácia da vacina*
Outros	=>	*Outros*
Sem_Opinião	=>	*Sem Opinião*

Visible if	
Q:	A:
Experiências passadas com vacinação?	- value => Negativa

Q: Você tem filhos? (*)

Type: choice

A: one of the following:

Sim	=>	*Sim*
Não	=>	*Não*

Q: Número de filhos (*)

Type: text

A: text input

Visible if	
Q:	A:
Você tem filhos?	- value => Sim

Q: Você aceitaria que o seu filho recebesse a vacina? (*)

Type: choice

A: one of the following:

Sim	=>	*Sim*
Não	=>	*Não*

Visible if	
Q:	A:
Você tem filhos?	- value => Sim

Q: (Se não), liste as razões que teria para que o seu filho não receba essa vacina? (*)

Type: choice_multiple

A: multiple answers possible:

Não_é_necessária	=>	*Não é necessária*
Não_funciona	=>	*Não funciona*
Membros_da_famíla_ou_ami_os_aconselharam_contra_isso	=>	*Membros da família ou amigos aconselharam contra isso*
Não_é_adequado_para_crianças	=>	*Não é adequado para crianças*
Medo_dos_efeitos_secundários	=>	*Medo dos efeitos secundários*
Eu_não_confio_nos_fabricantes	=>	*Eu não confio nos fabricantes*
Outros	=>	*Outros*

Visible if

Q:	A:
Você aceitaria que o seu filho recebesse a vacina?	- value => Não

Q: Compreendo perfeitamente sobre o que é este estudo e concordo em participar. Todas as informações que forneço podem ser usadas pelos pesquisadores para compreender melhor sobre as medidas preventivas para o coronavírus no meu país. (*)

Type: checkbox

A: checkbox
